# Supplementary material for: New Limits for Stability of Supercapacitor Electrode Material Based on Graphene Derivative
Source: Nanomaterials (Basel). 2020 Aug 31;10(9):1731. doi: 10.3390/nano10091731 (PMC7558132; doi:10.3390/nano10091731)
Supplement: Supplementary file 1 [file nanomaterials-10-01731-s001.pdf]

nanomaterials

Supplementary Materials

New Limits for Stability of Supercapacitor Electrode Material Based on Graphene Derivative

Veronika Šedajová [1,2,†], Petr Jakubec [1,*,†], Aristides Bakandritsos [1], Václav Ranc [1] and Michal Otyepka [1,2,*]

[1] Regional Centre of Advanced Technologies and Materials, Faculty of Science, Palacký University, Šlechtitelů 27, 78371 Olomouc, Czech Republic; veronika.sedajova@upol.cz (V.Š.); a.bakandritsos@upol.cz (A.B.); vaclav.ranc@upol.cz (V.R.)
[2] Department of Physical Chemistry, Faculty of Science, Palacký University,17. listopadu 1192/12, 77900 Olomouc, Czech Republic
* Correspondence: p.jakubec@upol.cz (P.J.); michal.otyepka@upol.cz (M.O.); Tel.: +42-(05)-85634474 (P.J. & M.O.)
† These authors contributed equally to this work.

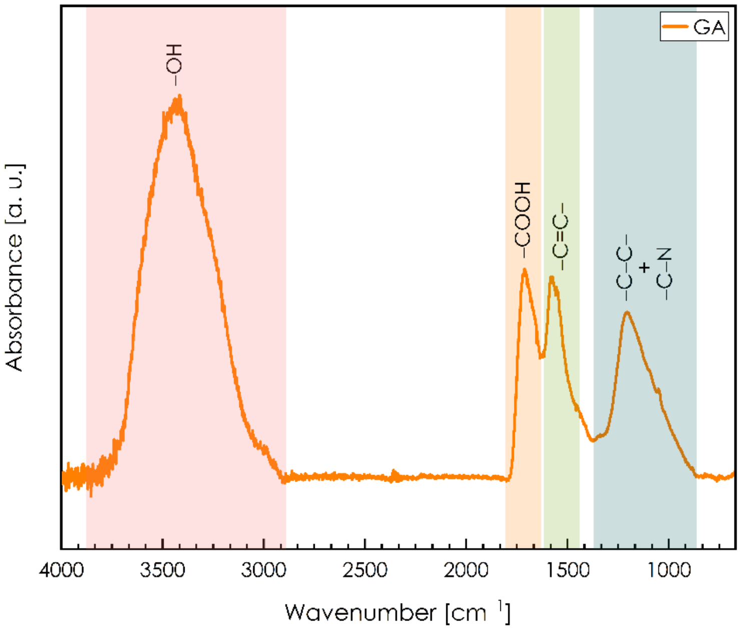

Figure S1. FTIR spectrum of graphene acid.

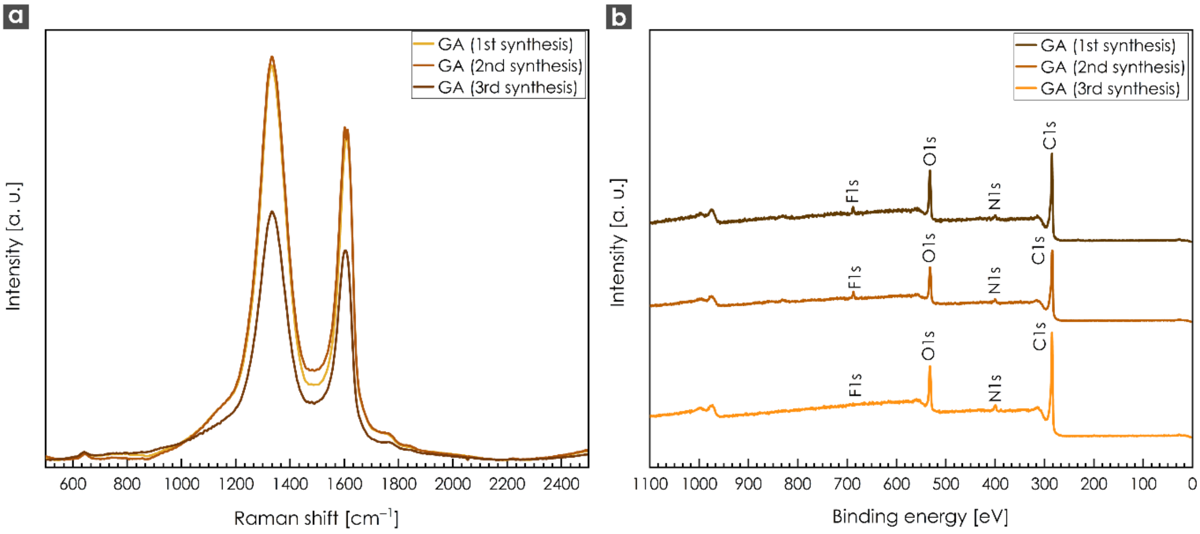

Figure S2. Comparison of (**a**) Raman and (**b**) XPS spectra of graphene acid from three independent syntheses.

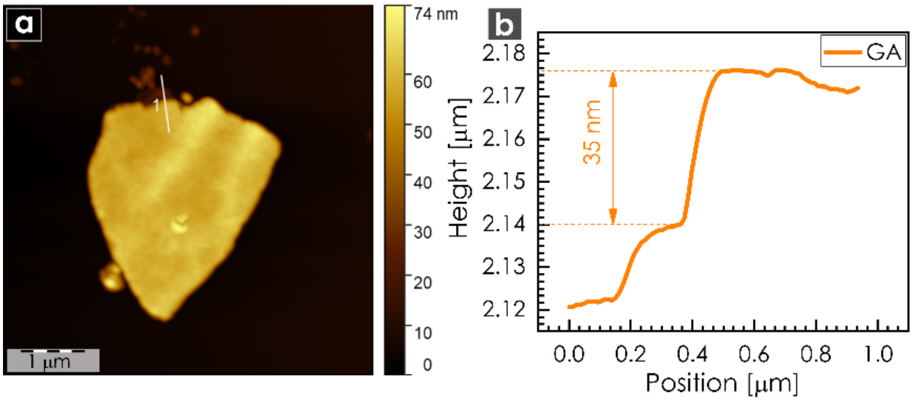

Figure S3. (**a**) AFM image of graphene acid and its corresponding high profile (**b**).

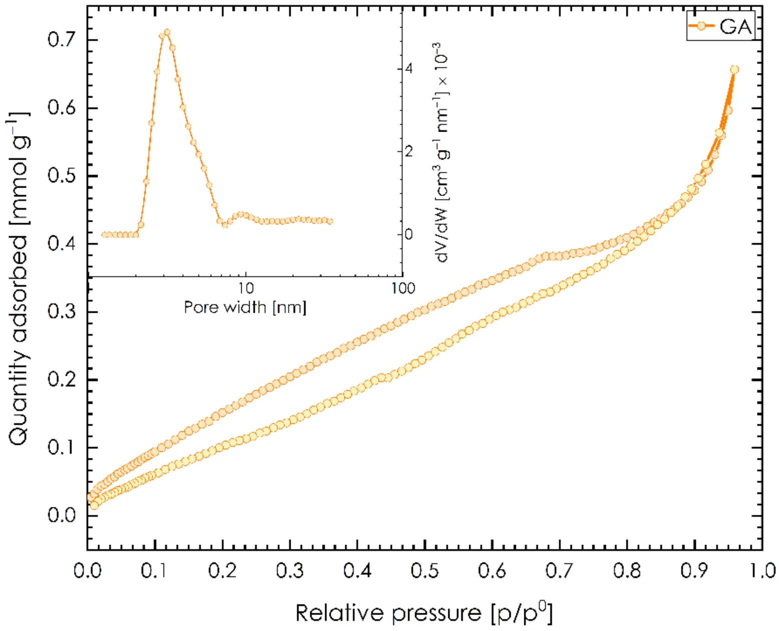

Figure S4. BET measurement of graphene acid with corresponding pore distribution (inset of Figure S4).

Table S1. Comparison of specific capacitance, energy density, power density, cyclic stability and capacitance retention in GA, commercial graphene from Ossila and other published comparable materials.

Reference	Cs (F/g)	E (Wh/kg)	P (kW/kg)	Cyclic Stability	Retention
[1]	96	12.25	0.5	10,000	91
[2]	53	6	0.5	10,000	92
[3]	367	16	0.8	1000	>100
[4]	141	19	0.5	20,000	90
[5]	261	36	0.5	200	93
Ossila graphene	92	12.8	0.5	-	-
**GA (1 A/g)**	**109**	**15**	**5**	**60,000**	**95**
**GA (10 A/g)**	**117**	**16**	**0.5**	**60,000**	**95**

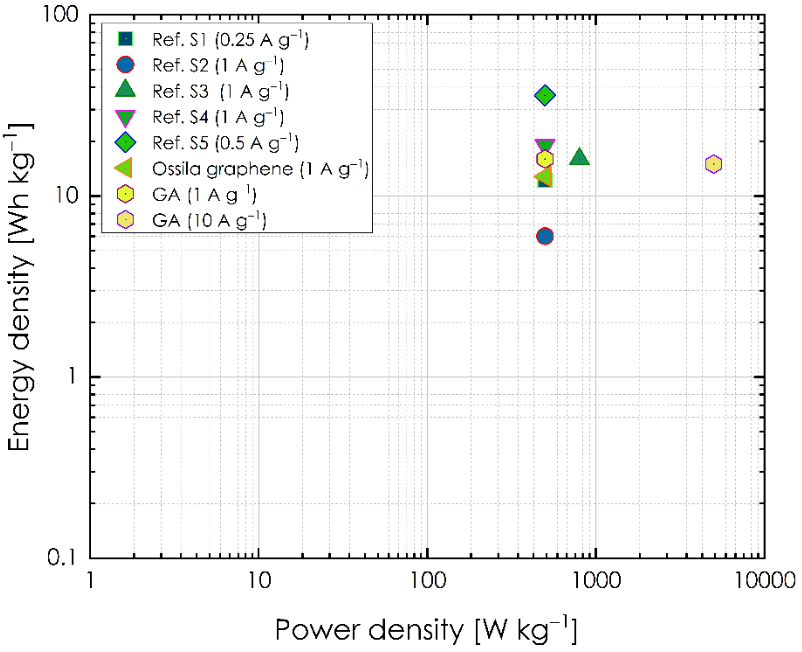

Figure S5. Ragone plot showing energy and power density of GA, commercial graphene and other comparable published materials. Specific capacitance was recalculated using the same metrics and formulas as in described in the main text of the manuscript, preferably from discharging time of GCD profiles. Energy density and power density values were calculated using the following equations: $E = (C_s \times 0.5 \times \Delta V^2)/3.6$; $P = 3600 \times (E/\Delta t)$, where E is the energy density (Wh kg^{-1}), P is the power density (W kg^{-1}), C_s has the meaning of specific capacitance (F g^{-1}), ΔV is the voltage window (V) and Δt is the discharge time (s).

Description of Trasatti's methodology

Using the Trasatti's methodology the total voltammetric charge, q_T, can be calculated from estimating charge at $\nu \rightarrow 0$ from the plot q^{-1} vs $\nu^{1/2}$ (Figure S6b). Electrochemical double-layer (EDLC) contribution can be obtained from double-layer charge, q_{DL}, which is estimated when $\nu \rightarrow \infty$ from the plot q vs. $\nu^{-1/2}$ (Figure S6a). Then, the pseudocapacitive contribution can be calculated as the difference between q_T and q_{DL} [6].

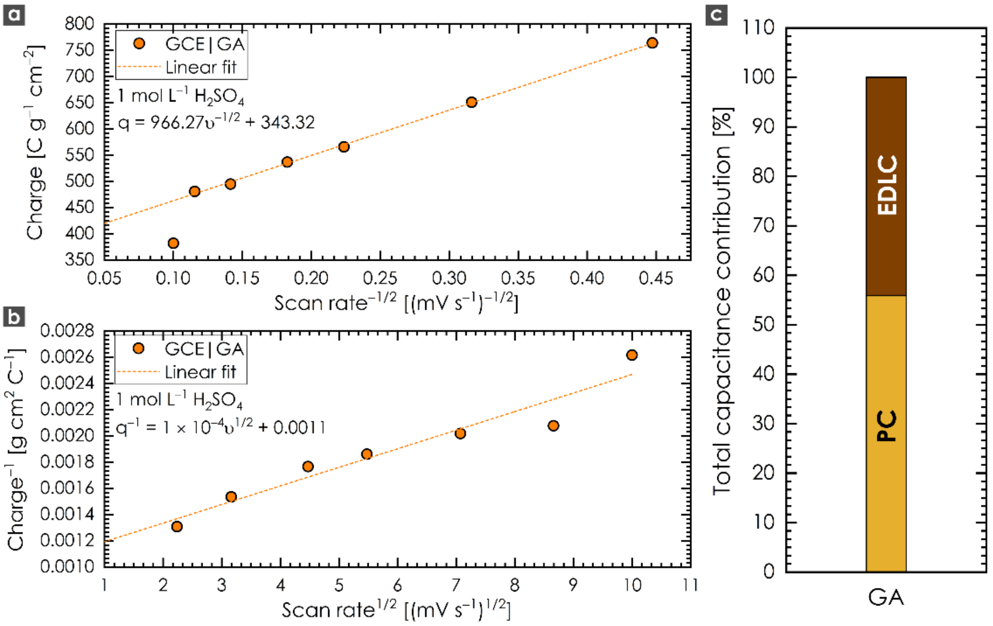

Figure S6. (**a**) Dependence of q on $v^{-1/2}$, (**b**) dependence of q^{-1} on $v^{1/2}$ for GA in 1 mol L^{-1} sulphuric acid electrolyte. (**c**) bar chart of the contribution of pseudocapacitance (PC) and electrochemical double-layer capacitance (EDLC) to the total capacitance.

References

1. Chen, Y.; Yan, Q.; Zhang, S.; Lu, L.; Xie, B.; Xie, T.; Zhang, Y.; Wu, Y.; Zhang, Y.; Liu, D. Buffering agents-assisted synthesis of nitrogen-doped graphene with oxygen-rich functional groups for enhanced electrochemical performance. *J. Power Sources* **2016**, *333*, 125–133.
2. Song, B.; Sizemore, C.; Li, L.; Huang, X.; Lin, Z.; Moon, K.; Wong, C.-P. Triethanolamine functionalized graphene-based composites for high performance supercapacitors. *J. Mater. Chem. A* **2015**, *3*, 21789–21796.
3. Johra, F.T.; Jung, W.-G. Hydrothermally reduced graphene oxide as a supercapacitor. *Appl. Surf. Sci.* **2015**, 357.
4. Xu, Y.; Chen, C.-Y.; Zhao, Z.; Lin, Z.; Lee, C.; Xu, X.; Wang, C.; Huang, Y.; Shakir, M.I.; Duan, X. Solution Processable Holey Graphene Oxide and Its Derived Macrostructures for High-Performance Supercapacitors. *Nano Lett.* **2015**, *15*, 4605–4610.
5. Liu, Y.; Zhang, Y.; Ma, G.; Wang, Z.; Liu, K.; Liu, H. Ethylene glycol reduced graphene oxide/polypyrrole composite for supercapacitor. *Electrochim. Acta* **2013**, *88*, 519–525.
6. Lee, J.-S.M.; Wu, T.-H.; Alston, B.M.; Briggs, M.E.; Hasell, T.; Hu, C.-C.; Cooper, A.I. Porosity-engineered carbons for supercapacitive energy storage using conjugated microporous polymer precursors. *J. Mater. Chem. A* **2016**, *4*, 7665–7673.

All RAW data are available as the .zip archive on Mendeley data repository (reserved DOI: 10.17632/mrhb67pzp6.1)

© 2020 by the authors. Submitted for possible open access publication under the terms and conditions of the Creative Commons Attribution (CC BY) license (http://creativecommons.org/licenses/by/4.0/).
